# Efferocytosis during Skeletal Muscle Regeneration

**DOI:** 10.3390/cells10123267

**Published:** 2021-11-23

**Authors:** Gaëtan Juban, Bénédicte Chazaud

**Affiliations:** Institut NeuroMyoGène, University of Lyon, INSERM U1217, CNRS UMR 5310, 8 Avenue Rockefeller, 69008 Lyon, France

**Keywords:** skeletal muscle regeneration, macrophages, efferocytosis, resolution of inflammation

## Abstract

Efferocytosis, i.e., engulfment of dead cells by macrophages, is a crucial step during tissue repair after an injury. Efferocytosis delineates the transition from the pro-inflammatory phase of the inflammatory response to the recovery phase that ensures tissue reconstruction. We present here the role of efferocytosis during skeletal muscle regeneration, which is a paradigm of sterile tissue injury followed by a complete regeneration. We present the molecular mechanisms that have been described to control this process, and particularly the metabolic control of efferocytosis during skeletal muscle regeneration.

## 1. Introduction

Macrophages control the inflammatory response after tissue injury. One of their primary duties is to cleanse the cell debris of the injured area through phagocytosis. Engulfment of cell debris has been described as non-phlogistic phagocytosis, then efferocytosis. Indeed, macrophages that have ingested dying cells act as dampeners of inflammation. Seminal studies demonstrated in vitro and in vivo that recognition and engulfment of apoptotic cells (through exposed phosphatidylserine) reduce the secretion of pro-inflammatory effectors by phagocyting macrophages to the benefit of the secretion of TGFβ and PGE2 [1,2,3]. Once efferocytosis is operated, these recovery macrophages orchestrate tissue repair, including parenchymal reorganization (either repair or regeneration depending on the capabilities of the parenchymal cells), angiogenesis, and matrix remodeling.

Here we present the role of efferocytosis during skeletal muscle regeneration, which is a paradigm of sterile tissue injury followed by a complete regeneration. Skeletal muscle is composed of myofibers which are syncytial cells with hundreds of myonuclei. These multinucleated cells are post-mitotic. Therefore, skeletal muscle regeneration relies on the properties of muscle stem cells (MuSCs), the satellite cells that implement a myogenic program, in coordination with the cells of their close environment to rebuild new functional myofibers. The presence of macrophages exerting phagocytotic functions in regenerating muscle was already described in 1957 [4]. In 1993, McLennan et al. identified by histological observations two subsets of macrophages with only one subset exhibiting an activity of phagocytosis of the damaged myofibers [5]. Since then, substantial advance has been made in our understanding of the role of macrophages and the pivotal role of efferocytosis that shifts their inflammatory profile and functions, delineating the transition from the pro-inflammatory phase of the inflammatory response to the recovery phase that ensures tissue reconstruction.

## 2. Pleiotropic Roles of Macrophages during Muscle Regeneration

### 2.1. Overview of Skeletal Muscle Regeneration

Adult skeletal muscle regeneration relies on the powerful properties of muscle stem cells that follow a cell-autonomous program of adult myogenesis, controlled by the sequential expression of Myogenic Regulatory transcription Factors (MRFs). Upon muscle damage, MuSCs exit the quiescent state (where they highly express Pax7) and start to proliferate (they down regulate Pax7 expression and express MyoD). Some myoblasts undergo asymmetric division giving rise to a small pool of cells that self-renew as satellite cells and to myogenic progenitors. The latter exit the cell cycle and enter into terminal differentiation, and become differentiated myocytes (extinguishing Pax7 expression and expressing Myogenin and MRF4). Finally, the differentiated cells fuse together to form myotubes, then grow into newly formed myofibers that maturate, reinnervate and recover their contractile function [6,7].

Although MuSCs exhibit a powerful cell-autonomous capability to implement myogenesis (isolated MuSCs spontaneously recapitulate adult myogenesis without external stimuli), it was shown that their close surrounding environment strongly impacts on their behavior, and therefore on the efficacy of the myogenesis process. One of the major components of this environment are immune cells and particularly macrophages, which are present in huge numbers (representing about 75% of hematopoietic cells in the cardiotoxin model). Macrophages accompany each step of the regeneration process, where they play a plethora of non-immune roles towards the cells present in the regenerating area [8].

Unless indicated, the results described in this review are coming from the widely used model of post-toxic injury muscle regeneration, where injury is provoked by the intramuscular injection of a toxin that damages almost the entire muscle (Tibialis Anterior or Gastrocnemius muscles) [6]. In this model, all myofibers are equally damaged and the regenerating process follows a highly reproducible kinetics where the sequential steps of muscle regeneration are precisely depicted. On top of that, this model is highly inflammatory, which is an advantage to study the inflammatory response.

### 2.2. Cell Interactions Established by Macrophages to Sustain Skeletal Muscle Regeneration

A few hours after a toxic (notexin or cardiotoxin) injury, pro-inflammatory circulating monocytes (Ly6C^pos^ CX3CR1^lo^ CCR2^pos^) enter the injured muscle through the CCL2/CCR2 axis and become pro-inflammatory macrophages (Ly6C^pos^ CX3CR1^lo^ CCR2^pos^ F4/80^lo^). Pro-inflammatory macrophages sustain the mounting of the inflammatory response and exert various functions towards cells residing in the muscle. This phase lasts no more than 3 days, and the resolution of inflammation takes place between day 2 and day 3 after injury (in the cardiotoxin model). The resolution of inflammation, operated by efferocytosis (see below) gives rise to anti-inflammatory/recovery/restorative macrophages (Ly6C^neg^ CX3CR1^hi^ CCR2^neg^ F4/80^hi^) that decrease the inflammatory response, and act on a variety of cell types to ensure tissue repair, angiogenesis, and matrix remodeling [8].

Cells that are known to be targeted by macrophages during muscle regeneration are MuSCs, endothelial cells (ECs) and fibroadipogenic precursors (FAPs), which are multipotent mesenchymal cells. During the first days after injury, i.e., during the pro-inflammatory phase, Ly6C^pos^ macrophages stimulate MuSC proliferation and inhibit their fusion, while inducing the apoptosis of a part of FAPs that have been activated by the injury. During the repair phase, recovery macrophages stimulate the late steps of myogenesis (MuSC terminal myogenic differentiation and fusion into multinucleated cells). Recovery macrophages also stimulate angiogenesis, which is coregulated with myogenesis. Finally, they promote the differentiation of FAPs into fibroblasts and their production of collagen; moreover, recovery macrophages secrete extracellular matrix components and enzymes involved in matrix remodeling [7,8,9].

A very interesting feature is the similarity between the sequences of macrophage activation profile in post-injury skeletal muscle regeneration in mouse and in human. Indeed, after an experimentally exercise-induced muscle injury of human healthy muscle, areas of regeneration discriminate early regenerating areas where pro-inflammatory macrophages and proliferating MuSCs coexist, from late regenerating areas where recovery macrophages are associated with differentiating MuSCs [10,11].

The powerful capacity of skeletal muscle to regenerate relies on this sequence of cell interactions that must be tightly ordered in time and space for an efficient functional recovery. In this aspect, the transition from the pro-inflammatory phase to the recovery phase is of utmost importance.

## 3. The Resolution of Inflammation, through Efferocytosis, Is Necessary for Skeletal Muscle Regeneration

### 3.1. Macrophage Inflammatory Profile Shift and the Resolution of Inflammation during Skeletal Muscle Regeneration

The presence of recovery macrophages that start the second phase of muscle regeneration could be due to either the recruitment of Ly6C^neg^ cells from the blood or the shift of the inflammatory profile of Ly6C^pos^ macrophages already present in the injured muscle. A series of evidence argue for the second hypothesis. First, depletion of circulating monocytes (using liposome-clodronate) from day 3 after injury does not impact the regeneration process [12]. Second, labeling circulating monocytes with fluorescent latex beads indicates that only Ly6C^pos^ enter into the injured muscle where they further lose Ly6C expression with time in the regenerating muscle [12]. Finally, in Nur77^KO^ animals, where Ly6C^neg^ monocytes are barely present in the circulation, both muscle regeneration and kinetics of macrophage inflammatory profile are similar to the wildtype [13]. Therefore, after a severe skeletal muscle injury, only Ly6C^pos^ inflammatory monocytes enter the injured muscle, and convert into recovery macrophages, proceeding to the resolution of inflammation, as has been shown in all injured tissues investigated so far.

Several molecular mechanisms were identified to control this key moment during skeletal muscle generation, delineating the stopping of the pro-inflammatory response and the start of the tissue repair. They include CREB-C/EBPβ, p38/MKP-1/AKT, meteorin-like/Stat3/IGF-1, RhoA-Rock1/Nfix and Annexin1/CaMMKβ/AMPK pathways. When those pathways are inhibited, muscle regeneration is strongly impaired [14,15,16,17,18,19]. Whether those pathways act in parallel or are interdependent (and to what extent) is still unclear. Given the crucial role of the resolution of inflammation in tissue repair, one may expect that redundant pathways operate to ensure the execution of this vital biological process. Interestingly, the last two mechanisms cited above have been directly linked to efferocytosis.

### 3.2. Efferocytosis-Induced Phenotypic Shift of Macrophages during Muscle Regeneration

First evidence of efferocytosis in skeletal muscle came from in vitro experiments where pro-inflammatory macrophages shift their inflammatory profile upon the ingestion of dead MuSCs [12]. Mechanical blockade of efferocytosis prevents the shift in cytokine secretion [12]. In vivo, Ly6C^neg^ recovery macrophages strongly express Secretory Leukocyte Peptidase Inhibitor (SLPI) [12], which expression is up-regulated upon efferocytosis, triggering the inhibition of neutrophil-derived proteases and dampening the inflammatory response [20]. In vivo evidence of efferocytosis during muscle regeneration came from seminal histological investigations, where pro-inflammatory macrophages are observed in the damaged areas [5,21,22]. The depletion of CX3CR1 in macrophages accelerates muscle regeneration and partly rescues the strong deficit observed in CCL2^KO^ animals (due to the deficit of monocyte entry into the injured muscle) [23]. CX3CR1 deficiency in macrophages increases their ApoE expression in vivo (ApoE being required for their efferocytic activity [24]) and their rewiring in recovery macrophages in the regenerating muscle [23].

**Figure 1:**
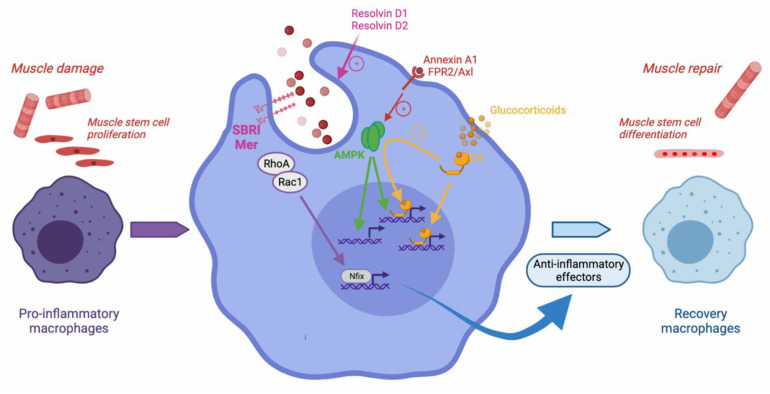
Efferocytosis during skeletal muscle regeneration. Upon muscle damage, pro-inflammatory monocytes enter the injured area and become pro-inflammatory macrophages that exert various properties such as stimulating the proliferation of muscle stem cells. Upon efferocytosis of muscle debris, they operate the resolution of inflammation and activate the expression of anti-inflammatory genes, and further shift into repair macrophages that stimulate myogenesis and the formation of new functional myofibers. Several molecular mechanisms have been involved in efferocytosis regulation during muscle regeneration. The scavenger receptor SBRI and Mer are required. RhoA/Rac1 inhibition leads to the expression of Nfix, a transcription factor involved in the expression of anti-inflammatory genes. The metabolic regulator AMPK is required for the efferocytosis-dependent resolution of inflammation. AMPK may be activated by the resolvin Annexin A1, through its FPR2/axl receptor. Resolvins D1 and D2 were also shown to increase efferocytosis and expedite muscle regeneration. Upon glucocorticoid stimulation, the glucocorticoid receptor activates AMPK and exerts both direct canonical and indirect non-canonical anti-inflammatory effects. The non-canonical action stimulates the expression of genes involved in phagocytosis and efferocytosis, therefore, boosting the resolution of inflammation.

Three recent studies have shown the direct requirement of mechanisms involved in efferocytosis as necessary for muscle regeneration (Figure 1). Scavenger receptor class B1 (SRB1) is a phosphatidylserine receptor, and as such, recognizes apoptotic cells. SRB1 in macrophages, through ERK1/2 activation, is required for the efferocytosis of dead myoblasts in vitro and for the acquisition of the recovery phenotype in vitro and in vivo during muscle regeneration [25]. As a consequence, lack of SRB1 in macrophages is associated with a strongly impaired muscle regeneration due to a deficit of the transition of macrophages towards the recovery phenotype in vivo [25]. Similarly, mice deficient for Mer (one of the TAM receptors that are involved in efferocytosis) show impaired muscle regeneration associated with a delayed resolution of inflammation by macrophages. In vitro experiments show a deficit of Mer^KO^ macrophages for engulfment of dead myoblasts. In this study, using whole body KO animals, Mer deficiency was shown to impact both the resolution of inflammation and myogenesis [26]. In the same vein, inhibition of the RhoA-ROCK1 pathway, which increases phagocytosis, triggers the expression of the transcription factor Nfix during skeletal muscle regeneration. Nfix deficient macrophages are unable to adopt the recovery phenotype in vitro and in vivo, therefore impeding on muscle regeneration [19]. These three studies demonstrate that the efferocytic mechanism is a major event leading to the shift of macrophage inflammatory phenotype and the resolution of inflammation necessary for skeletal muscle regeneration.

The in vitro studies described above have used various ways to induce myoblast (C2C12 cell line or primary human and mouse myoblasts) death including H_2_O_2_ and H_2_O treatment, leading to mainly apoptotic and necrotic cells in the culture, respectively. Although the mechanisms by which macrophages recognize necrotic cells must be investigated, the shift of macrophage inflammatory phenotype was observed with both apoptotic and necrotic myoblasts.

## 4. The Link between Efferocytosis and Cellular Metabolism

The role of cellular metabolism on macrophage inflammatory status and function has been increasingly studied during recent years [27,28]. The resolution of inflammation is characterized by a metabolic rewiring from glycolysis in pro-inflammatory macrophages towards oxidative phosphorylation in anti-inflammatory macrophages [27,29]. Efferocytosis is a high energy demanding process, as it requires profound modifications within the efferocyte such as a major cytoskeleton reorganization to form the phagocytic cup and the phagosomes. However, the link between efferocytosis and metabolic rewiring is still poorly characterized. In particular, it is still unclear whether the metabolic reprogramming is required to sustain the efferocytic process, or if efferocytosis triggers the rewiring which is necessary to drive the resolution of inflammation.

### 4.1. Metabolic Pathways Linked to Efferocytosis

A recent study [30] demonstrated the requirement of the glycolytic pathway for the efferocytic process in macrophages, using an in vitro model of LR73 hamster phagocytes engulfing apoptotic human Jurkat cells. Transcriptomic analyses identified a specific set of solute carrier (SLC) family members regulated by apoptotic cell engulfment, including the up-regulation of the SLC2A1 glucose transporter, whereas SLCs involved in oxidative phosphorylation and fatty acid oxidation are down-regulated. This is associated with an increased glucose uptake and increased glycolysis. Consequently, glycolysis inhibition or genetic depletion of Slc2a1 decreases macrophage efferocytic capacity. Unexpectedly, this study also highlights the role of glycolysis in promoting the resolution of inflammation. Indeed, apoptotic cells engulfment up-regulates the SLC16A1 transporter which mediates the secretion of lactate, the end product of glycolysis. Interestingly, the authors suggested that this lactate release favors an anti-inflammatory environment contributing to the resolution of inflammation as efferocyte-derived conditioned medium induces the expression of IL10 and TGFβ anti-inflammatory genes by naive murine macrophages in vitro.

Mitochondria have also been shown to be important for efferocytosis. For instance, efferocytosis up-regulates the levels of the mitochondrial membrane uncoupling protein (UCP)2 in an in vitro model of apoptotic thymocytes engulfed by LR73 phagocytes, while genetic depletion of *Ucp2* in mouse reduces macrophage phagocytic activity in vivo in a model of dexamethasone-induced thymocyte apoptosis [31]. Similarly, altered macrophage phagocytic capacity (of apoptotic thymocytes) is observed in mice depleted for dynamin-related protein (Drp)1, a mediator of mitochondrial fission that is required for phagosome formation, through the calcium release from the endoplasmic reticulum [32].

More recently, a molecular network linking efferocytosis and the production of the IL-10 anti-inflammatory cytokine, through the stimulation of mitochondria activity, was identified in murine macrophages after myocardial infarction [33]. Metabolomics analyses showed that efferocytosis induces fatty acid oxidation and the NAD+ production, involving electron transport by the Rieske iron-sulfur protein (RISP). NAD+ then activates the sirtuin (SIRT1) deacetylase that in turn favors the binding of pre-B cell leukemia transcription factor (PBX)1 on the IL10 gene promoter and stimulates its expression. Importantly, disruption of this molecular network by *Risp* depletion in macrophages results in an impaired resolution of inflammation after myocardial infarction in mouse. Although these results appear in contradiction with the study by Morioka et al., they may reflect the requirement of a sequential activation of different metabolic pathways to sustain the efferocytic process and to promote the resolution of inflammation. Thus, exhaustive in vivo kinetics analyses are required to accurately characterize this metabolic sequence.

### 4.2. Disposal/Recycling of Metabolites from Engulfed Cells

Upon efferocytosis, macrophages ingest bodies that can be as large as them, thus bringing a strong metabolite load that needs to be externalized or catabolized to restore the macrophage metabolic homeostasis. In vitro studies have shown that lipids from the membrane of the engulfed cells accumulate into macrophages and generate ligands for liver X receptor (LXR) and peroxisome proliferator-activated receptor (PPAR) family members, which are nuclear receptors regulating lipid export and metabolism [34]. Engulfment of apoptotic cells was shown to increase cholesterol efflux by macrophages, through the activation of LXR and PPARγ, and the subsequent up-regulation of the ATP-binding cassette (ABC)A1 membrane transporter [35]. During muscle regeneration, PPARγ is not required for the efferocytosis of dead myoblasts *per se*, but its expression is mandatory in macrophages for the subsequent acquisition of the recovery phenotype [36]. Efferocytosis of dead myoblasts also activates another PPAR family member, PPARδ (aka PPARβ) [37], which has been linked to fatty acid oxidation in muscle cells [38].

Another mechanism to restore the efferocyte metabolic balance was recently identified. Zhang et al. showed in murine macrophages that the intensity of the IL-10 production triggered by efferocytosis varies depending on the membrane content of the engulfed cells. Although the engulfment of inert beads does not stimulate mitochondrial fatty acid oxidation or IL-10 synthesis, increased fatty acid levels in the membrane of the ingested apoptotic cells results in an increased IL-10 production by efferocytic macrophages [33]. This suggests that efferocytes can use the metabolites from the engulfed bodies to fuel the molecular network that drives the resolution of inflammation.

## 5. Immunometabolism, Efferocytosis, and Skeletal Muscle Regeneration

During skeletal muscle regeneration, it was shown that the metabolic profile of macrophages evolves with time. Indeed, RNAseq of isolated Ly6C^pos^ and Ly6C^neg^ macrophages at various time points of regeneration indicates that their metabolic change, from a glycolytic to an oxidative metabolism, shortly precedes the shift of their inflammatory profile. Moreover, longitudinal analysis of the cell profile along the regeneration process indicates that more than expressing or not the Ly6C marker, the time after injury is the driving force of the metabolic and inflammatory changes in macrophages [9,39].

### 5.1. Efferocytosis and Metabolic Circuits in Macrophages during Muscle Regeneration

One of the main metabolic sensors of the cells, 5′AMP kinase (AMPK), is activated in macrophages upon efferocytosis, triggering microtubule and actin reorganization [40,41]. On the other hand, AMPKα1 deficient macrophages (alpha1 is the only catalytic subunit expressed in macrophages) show a lower phagocytic activity, whatever their inflammatory status [16] while activation of AMPK increases this phagocytic activity [40]. As discussed above, it is difficult to assess if some metabolic requirements are needed for efferocytosis or if efferocytosis itself also rewires the metabolic circuits in macrophages. Both are probably true.

During skeletal muscle regeneration, AMPK activation is required in macrophages for their acquisition of the recovery phenotype in vitro and in vivo [16]. Intriguingly, the shift is efferocytosis-dependent since AMPK deficient macrophages do not rewire their pro-inflammatory profile in the presence of dead myoblasts. Therefore, AMPK activation and phagocytosis act together to trigger the macrophage phenotypic shift at the time of resolution of inflammation. Upstream AMPK activation, its activator Ca2+/calmodulin-dependent protein kinase kinase-beta (CaMKKbeta) is required for the efferocytosis-dependent shift of macrophage inflammatory profile [16]. Apoptotic cell recognition induces both acute and sustained calcium fluxes within macrophages, and the molecules required for calcium flux are essential for engulfment, partly regulated by mitochondrial metabolism [32,42,43]. Thus, during skeletal muscle regeneration, efferocytosis is a major event for the resolution of inflammation, associated with specific activation of metabolic regulators associated with mitochondria function.

### 5.2. Modulation of Metabolism and Efferocytosis during Muscle Regeneration

Although skeletal muscle is highly powerful for regeneration, there is some interest to expedite the regeneration process, for instance in the case of infection that disrupts the inflammatory response [44], in the case of repeated injuries that alter the kinetics of regeneration [45] or, in practice, after exercise-induced muscle damage, especially for elite athletes.

One of the most prescribed anti-inflammatory compounds are glucocorticoids. In vitro, glucocorticoids shift macrophages towards a recovery phenotype, which exert their repair function on MuSCs [46]. The glucocorticoid dexamethasone stimulates the phagocytic activity of macrophages, including efferocytosis [46,47,48,49]. A recent investigation showed that dexamethasone activates AMPK in macrophages and that AMPK activation is required for the anti-inflammatory effect of dexamethasone. AMPK deficient macrophages do not acquire the recovery phenotype and associated functions upon dexamethasone treatment [50]. Transcriptomic analyses identified a non-canonical action of glucocorticoids that is dependent of AMPK through the control of the expression of genes involved in phagocytosis and efferocytosis. This was confirmed functionally in vitro and in vivo where AMPKα1-deficient macrophages do not increase their efferocytic activity upon glucocorticoid treatment during muscle regeneration. In vivo, administration of dexamethasone before the onset of the resolution of inflammation (i.e., 3 days in the cardiotoxin model) alters skeletal muscle regeneration, confirming that a too early shift of macrophages is detrimental for muscle repair [14,17]. On the contrary, dexamethasone expedites muscle regeneration when administrated from the onset of the resolution of inflammation [50].

Interestingly, Annexin A1, which is a pro-resolving molecule, is required for the increased macrophage phagocytic activity induced by dexamethasone [51,52]. Annexin A1 treatment increases macrophage efferocytosis. During skeletal muscle regeneration, Annexin A1, which is expressed by neutrophils and pro-inflammatory macrophages, activates AMPK in macrophages through its FPR2/AXL receptor, driving the resolution of inflammation [15]. The use of resolvins seems to be an interesting approach to expedite skeletal muscle regeneration while avoiding the adverse effects observed with classical anti-inflammatory drugs when administered early after the injury [53]. Indeed, treating mice with Resolvin D1 or Resolvin D2 improves skeletal muscle regeneration, in association with an increased phagocytosis and an efficient shift of macrophage inflammatory profile [39,54].

## 6. Conclusions

Efferocytosis is a key event that ensures an efficient skeletal muscle regeneration, to end the pro-inflammatory response and to start the repair process, as in many other tissues. There are many questions that remain to be investigated. One is to decipher the heterogeneity of macrophages during skeletal muscle regeneration to establish whether some subsets of macrophages are specifically in charge of efferocytosis and what are their specific responses during the process. Additionally, metabolic rewiring of macrophages upon efferocytosis deserves further investigation to establish if there are some specificities in the muscle tissue. Finally, as nicely exposed by Rothlin et al. [55], efferocytosis integrates the type and/or activation state of the efferocyte(s), the type and number of dead cells, and the cues present in the close environment. Dissection of this integrated process will identify the specific features of efferocytosis during muscle regeneration, and will identify targets for improving the resolution of inflammation in chronic muscle injuries or in an infection context.

## Data Availability

Not applicable.
